# Global trends, translational gaps, and emerging research directions in *Pleurotus* spp. bioactivity research: a 25-year scientometric analysis

**DOI:** 10.3389/ffunb.2026.1853358

**Published:** 2026-07-27

**Authors:** Kevin Smith P. Cabuhat, Jamil Allen G. Fortaleza, Johnalie N. Cabalbag, Christian Joseph N. Ong, Jowi Tsidkenu Pili Cruz, Nicole Rajah C. del Rosario, Mary Ylane S. Lee, Jose Jurel M. Nuevo, Joel G. Matamis, Jose Edwardo R. Mamaat, Amelda C. Libres, Rich Milton R. Dulay

**Affiliations:** 1 Basic Education Department, La Consolacion University Philippines, Malolos, Bulacan, Philippines; 2 Department of Biology, College of Science, De La Salle University, Manila, Philippines; 3 National University, Sampaloc, Manila, Philippines; 4 Department of Biology, Bulacan State University, Malolos, Bulacan, Philippines; 5 College of Medical Laboratory Science, Our Lady of Fatima University, Valenzuela, Philippines; 6 School of Medical Laboratory Sciences, St. Dominic College of Asia, Cavite, Philippines; 7 Department of Medical Technology, Far Eastern University, Manila, Philippines; 8 College of Medical Laboratory Science, Liceo de Cagayan University, Cagayan de Oro, Philippines; 9 Center for Tropical Mushroom Research and Development, Central Luzon State University, Science City of Muñoz, Nueva Ecija, Philippines

**Keywords:** *Pleurotus*, collaboration networks, circular bioeconomy, scientometric analysis, thematic evolution

## Abstract

**Introduction:**

*Pleurotus* spp. have attracted significant scientific interest for their diverse bioactive compounds and broad pharmacological potential. Despite the rapid growth of the literature, a comprehensive scientometric synthesis of this research landscape remains limited.

**Methods:**

This study used a systematic scientometric approach to analyze global research trends in *Pleurotus* bioactivity from 2000 to 2025. Key analyses included publication productivity, citation dynamics, co-authorship and country-level collaboration networks, keyword co-occurrence mapping, and thematic evolution.

**Results:**

The results reveal relatively stable publication output accompanied by a marked increase in citation impact after 2012, indicating growing intellectual consolidation and global recognition of the field. China and India dominate research productivity, whereas citation influence is more widely distributed, highlighting disparities between output and impact. Thematic evolution shows a shift from early studies on cultivation and compositional profiling toward advanced research in molecular bioactivity, gut microbiota modulation, enzymatic biotechnology, and circular bioeconomy applications. Emerging directions include precision nutraceutical development, optimization of extraction and fermentation processes, and agro-industrial waste valorization. Despite increasing interdisciplinary integration and strong preclinical evidence, collaboration networks remain partially fragmented, and translation into clinical and industrial applications remains limited.

**Discussion:**

These findings indicate that while research on *Pleurotus* has reached a stage of disciplinary maturity, significant gaps persist in standardization, clinical validation, and scalability. Addressing these challenges will require integrative omics approaches, strengthened global collaboration, and translational frameworks to bridge the gap between laboratory discoveries and real-world applications.

## 1 Introduction

*Pleurotus* spp., commonly referred to as oyster mushrooms, represent a taxonomically diverse and ecologically adaptable genus of basidiomycete fungi widely distributed across tropical and subtropical ecosystems, where they play a critical role in lignocellulosic decomposition and nutrient cycling (Phonemany et al., 2021; Febnteh et al., 2025; Raman et al., 2021). Comprising approximately 208 recognized species, the genus exhibits substantial genetic, morphological, and ecological heterogeneity, indicating its evolutionary adaptability and broad environmental niche (Flores et al., 2023). Beyond their ecological significance, *Pleurotus* species have emerged as one of the most economically important cultivated mushrooms globally, accounting for nearly 25% of total mushroom production. This widespread cultivation underscores their dual importance as both a food resource and a biotechnological platform, particularly in regions across Asia, Europe, and North America (Raman et al., 2021). In parallel, their enzymatic machinery enables applications in lignocellulosic degradation, waste valorization, and environmental bioremediation, positioning *Pleurotus* as a key organism at the intersection of sustainable biotechnology and circular bioeconomy systems (Gregori et al., 2007).

The increasing scientific attention toward *Pleurotus* spp. is primarily driven by their extensive repertoire of bioactive compounds, including polysaccharides, phenolics, and secondary metabolites with demonstrated pharmacological properties. These compounds exhibit a broad spectrum of biological activities, such as antioxidant, anti-inflammatory, antimicrobial, and anticancer effects, thereby establishing *Pleurotus* as a promising candidate for nutraceutical development and therapeutic innovation (Mazumder et al., 2025). Accumulating evidence further highlights their role in mitigating chronic metabolic disorders, including diabetes, obesity, cardiovascular diseases, and gastrointestinal dysfunction, reinforcing their relevance within functional food and preventive medicine frameworks (Ajanaku et al., 2022; Zhang et al., 2020). Moreover, emerging studies indicate that *Pleurotus-*derived bioactives may influence neuroprotective pathways through modulation of oxidative stress and inflammatory signaling, suggesting potential applications in neurodegenerative disease management, including Alzheimer’s disease (Zhang et al., 2020). Collectively, these findings reflect a progressive shift from nutritional characterization toward mechanistic and translational research domains.

Despite the rapid expansion of literature, the intellectual structure, thematic evolution, and translational trajectory of *Pleurotus* research remain insufficiently integrated. Existing studies often address bioactivity, biotechnology, or pharmacological potential in isolation, limiting a comprehensive understanding of how these domains converge to shape the field’s development. Scientometric analysis offers a robust quantitative approach to address this gap by systematically mapping publication outputs, citation networks, collaborative structures, and thematic clusters over time. Beyond descriptive metrics, advanced bibliometric and network-based analyses enable the identification of emerging research frontiers, knowledge gaps, and structural imbalances within scientific ecosystems (Konur, 2020; Chen et al., 2025; Obajemu et al., 2012). When coupled with thematic interpretation, these approaches provide critical insights into how research domains evolve from foundational discovery toward applied and translational outcomes (Aksenova and Tarkhov, 2023).

In this context, the present study provides a comprehensive and integrative scientometric assessment of global research on the bioactivity and pharmacological potential of *Pleurotus* spp. over the past 25 years (2000-2025). Specifically, this study aims to (i) map the intellectual and collaborative structure of the field through publication, citation, and network analyses, (ii) characterize the thematic evolution and emerging research frontiers, and (iii) critically evaluate the alignment between research productivity and translational progression toward clinical, industrial, and sustainability applications.

## 2 Methodology

### 2.1 Data source and search strategy

A bibliometric analysis was performed to assess global research trends concerning the bioactivity and pharmacological potential of *Pleurotus* species. The Scopus database was chosen due to its comprehensive coverage of peer-reviewed biomedical and interdisciplinary literature. The search was limited to English-language journal articles published from 2000 to 2025. The search strategy incorporated species-specific terms (such as *Pleurotus ostreatus*, *P. eryngii*, *P. pulmonarius, P. sajor-caju, P. florida, and P. citrinopileatus*) in combination with keywords related to bioactivity and pharmacology. The complete search query used in Scopus was as follows:

TITLE-ABS-KEY (Pleurotus W/2 (species OR spp.) OR “Pleurotus ostreatus” OR “Pleurotus eryngii” OR “Pleurotus pulmonarius” OR “Pleurotus sajor-caju” OR “Pleurotus florida” OR “Pleurotus citrinopileatus”) AND TITLE-ABS-KEY (“bioactiv*” OR “pharmacological activity” OR “biomedical application*” OR “therapeutic potential” OR “*in vitro*” OR “*in vivo*” OR “mechanism of action”) AND PUBYEAR > 1999 AND PUBYEAR < 2026 AND (LIMIT-TO (SRCTYPE, “j”)) AND (LIMIT-TO (PUBSTAGE, “final”)) AND (LIMIT-TO (DOCTYPE, “ar”)) AND (LIMIT-TO (LANGUAGE, “English”)).

### 2.2 Screening and eligibility criteria

**Figure 1:**
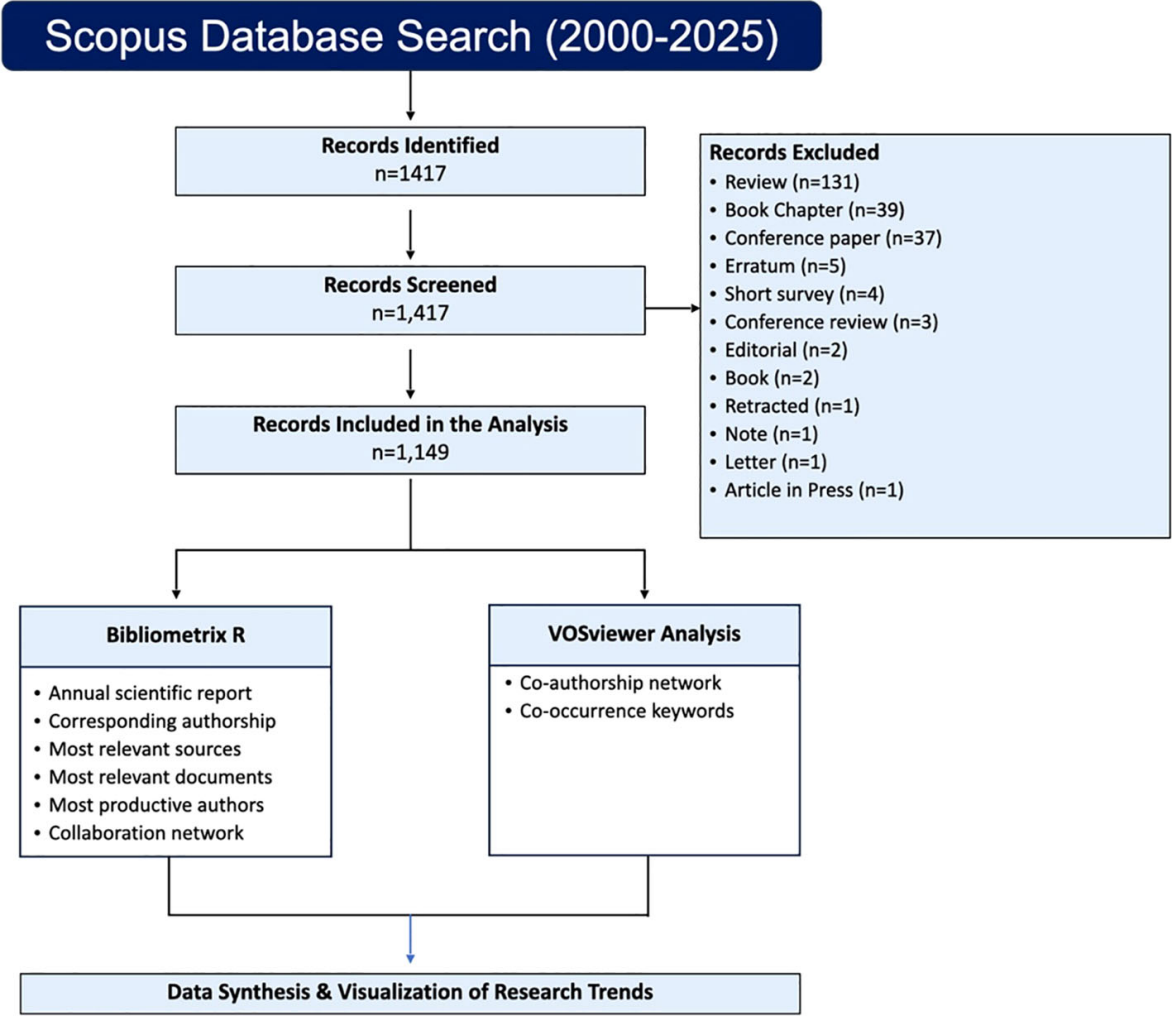
Workflow of the bibliometric analysis and data processing pipeline.

The analysis included only final journal articles published in English. Non-primary literature, such as conference papers, editorials, reviews, book chapters, notes, and other forms of gray literature, was excluded. Studies unrelated to the bioactivity, pharmacological properties, therapeutic applications, or mechanisms of action of *Pleurotus* species were also excluded to enhance dataset relevance and analytical precision. **Figure 1** provides an overview of the study workflow, encompassing data retrieval, screening, eligibility assessment, and bibliometric analysis.

### 2.3 Bibliometric analysis framework

The bibliometric analysis followed the framework established by Donthu et al. (2021), integrating performance analysis and science mapping to assess the development of research on *Pleurotus* spp. bioactivity. Performance analysis evaluated research productivity and scientific impact across publications, countries, journals, authors, institutions, and cited documents. Annual scientific production and cumulative growth trends were analyzed to characterize the field’s temporal evolution. Citation-based indicators, including total publications (TP), total citations (TC), average citations per publication (ACP), and h-index, were used to measure scholarly influence. Country contributions were determined by corresponding author affiliations, and international collaboration was assessed using Single Country Publications (SCP) and Multiple Country Publications (MCP). The analysis identified the most productive journals, influential authors, institutions, countries, and highly cited documents to establish the field’s intellectual foundations and scientific impact.

### 2.4 Science mapping and network visualization

The dataset was analyzed using Biblioshiny (Bibliometrix R package) and VOSviewer version 1.6.20 (Centre for Science and Technology Studies [CWTS], Leiden University, Netherlands). Biblioshiny facilitated the assessment of annual scientific production, citation patterns, leading authors, institutional productivity, source journals, trend topics, thematic maps, and thematic evolution. Science mapping was conducted using VOSviewer to examine the social and conceptual structures of the research domain, following approaches commonly used in recent bibliometric studies (Akin et al., 2025). Co-authorship networks were constructed to evaluate collaboration patterns among authors, institutions, and countries. Keyword co-occurrence analysis of author keywords identified major thematic areas and their interrelationships within the literature. Network, overlay, and density visualizations were generated to reveal collaboration clusters, thematic structures, and emerging research directions. Clustering functions in VOSviewer grouped closely related authors, institutions, countries, and keywords into distinct collaborative and thematic clusters. In the resulting maps, node size indicated frequency of occurrence or productivity, link thickness represented the strength of relationships, and colors denoted cluster membership or temporal characteristics in overlay visualizations.

## 3 Results

### 3.1 Annual number of publications and cumulative growth curve

**Figure 2:**
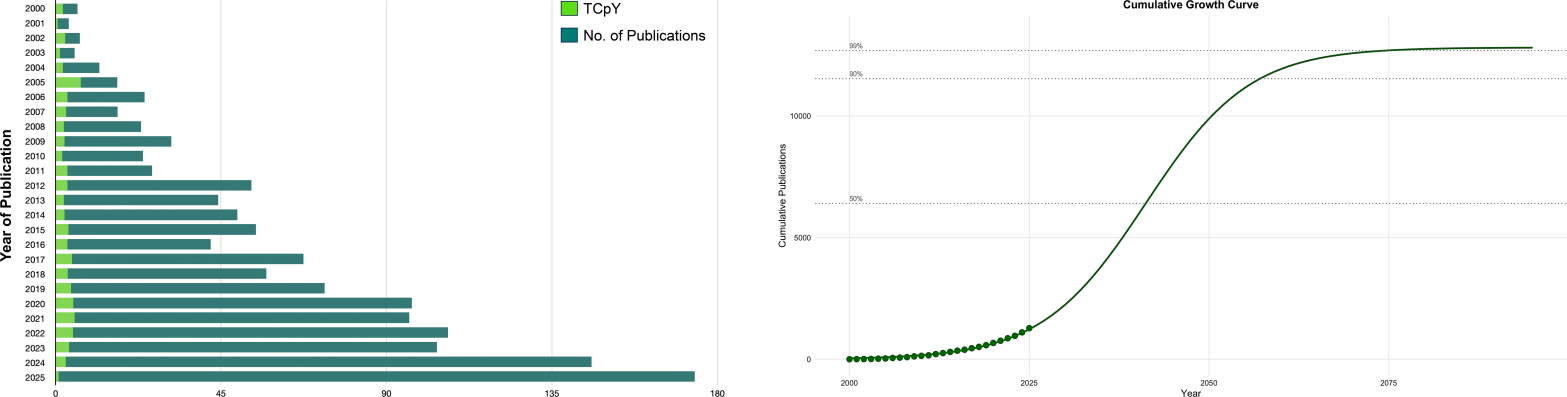
Cumulative growth and scientometric trends in *Pleurotus* spp. bioactivity research.

The bibliometric analysis demonstrates a dynamic yet progressively stabilizing trajectory in *Pleurotus* spp. bioactivity research, characterized by a divergence between publication output and citation impact. As illustrated in **Figure 2**, the annual number of publications shows a steady increase from the early 2000s, followed by a relatively stable output in recent years, indicating sustained research activity rather than exponential growth. In contrast, total citations per year (TCpY) exhibit a pronounced surge beginning around 2012, reflecting delayed recognition and increasing scholarly impact of earlier foundational studies. The cumulative growth curve further delineates this progression, with an initial exploratory phase (2000–2010) marked by gradual accumulation of publications, transitioning into a rapid expansion phase (2011–2020) driven by intensified research activity and collaborative efforts, and ultimately approaching a plateau in recent years. This plateau suggests a maturation of the field, where knowledge production stabilizes while impact continues to consolidate.

### 3.2 Leading and collaborating countries

**Figure 3:**
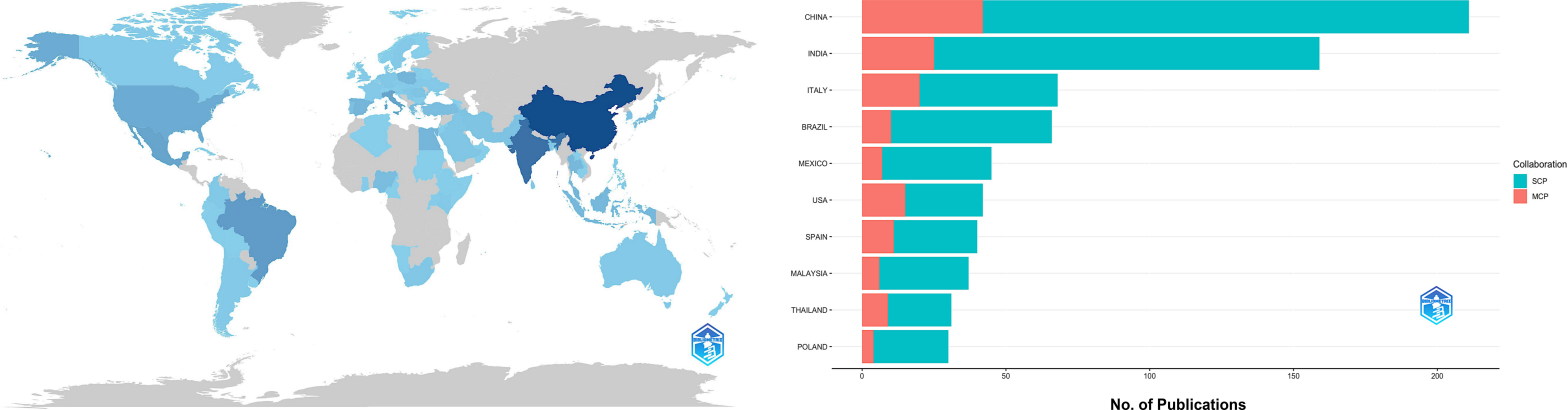
Global publication map showing research productivity by country and a bar chart presenting the top 10 contributing countries according to publication output.

The global landscape of *Pleurotus* research exhibits notable geographic variation, reflecting differences in productivity and collaboration. Mapping national contributions clarifies how domestic research strength and international partnerships shape the field’s development. This analysis identifies leading countries and emerging hubs over a 25-year period (**Figure 3**). China (n=230) and India (n=211) dominate, reflecting highly active scientific communities. The United States (n=84) and Italy (n=81) contribute significantly, while emerging hubs such as Brazil (n=79), Mexico (n=61), Egypt (n=44), and Nigeria (n=37) illustrate growing global engagement. These collaborations show how the field has grown through a mix of local expertise and international partnerships. Further insight into collaboration patterns is provided by a corresponding authorship analysis that distinguishes between Single Country Publications (SCP) and Multiple Country Publications (MCP). The results indicate that leading contributors such as China and India exhibit a predominance of SCP, suggesting strong domestic research capacity and well-established internal networks that sustain high publication output independently. In contrast, countries such as the United States, Italy, and Brazil demonstrate a relatively higher proportion of MCP, reflecting greater engagement in international collaborations and cross-border knowledge exchange.

### 3.3 Most Relevant Sources and cited documents

The bibliometric analysis reveals a structurally diverse yet hierarchically organized publication landscape in *Pleurotus* spp. bioactivity research, where productivity and citation impact are distributed unevenly across journals and seminal studies. As shown in **Table 1**, the *International Journal of Medicinal Mushrooms* ranks first in total publications (TP = 71), underscoring its role as a specialized outlet for mushroom-focused research. However, its comparatively lower CiteScore (2.9) and average citations per publication (ACP = 18.99) suggest that while it serves as a primary dissemination platform, its global citation influence is relatively limited. In contrast, broader, high-impact journals such as *Food Chemistry* (CiteScore = 18.3; ACP = 63.52) and *Carbohydrate Polymers* (CiteScore = 24.0; ACP = 56.64) exhibit significantly higher citation performance despite lower publication counts, indicating that studies published in these venues achieve greater visibility and scientific impact. This pattern highlights a dual publication dynamic in the field: specialized journals sustain research volume, whereas interdisciplinary journals amplify research influence. Similarly, the strong citation metrics of the *International Journal of Biological Macromolecules* (TC = 1,495; ACP = 39.34) and *Food and Function* (ACP = 35.06) reflect the increasing research emphasis on polysaccharides, macromolecular bioactives, and functional food applications of *Pleurotus* spp., signaling a shift toward biochemical and nutraceutical-oriented investigations.

**Table 1:** Top 10 most relevant sources publishing *Pleurotus* spp.

Rank	Journal title	CiteScore 2024	Publisher	TP	TC	ACP	h-index
1^st^	International Journal of Medicinal Mushrooms	2.9	Begell House	71	1,348	18.99	20
2^nd^	International Journal of Biological Macromolecules	10.3	Elsevier	38	1,495	39.34	21
3^rd^	Food Chemistry	18.3	Elsevier	33	2,096	63.52	22
4^th^	Molecules	8.6	Multidisciplinary Digital Publishing Institute (MDPI)	22	743	33.77	15
5^th^	Foods	8.7	Multidisciplinary Digital Publishing Institute (MDPI)	19	391	20.58	10
6^th^	Food and Function	10.5	Royal Society of Chemistry	16	561	35.06	11
7^th^	LWT	13.6	Elsevier	15	497	33.13	13
8^th^	Journal of Agricultural and Food Chemistry	9.3	American Chemical Society	14	558	39.86	11
9^th^	Animal Feed Science and Technology	6.0	Elsevier	11	429	39.00	8
9^th^	Carbohydrate Polymers	24.0	Elsevier	11	623	56.64	11
9^th^	PLOS One	5.4	Public Library of Science	11	306	27.82	9
10^th^	World Journal of Microbiology and Biotechnology	6.7	Springer Nature	9	166	18.44	6
10^th^	Journal of the Science of Food and Agriculture	7.9	John Wiley & Sons	9	211	23.44	8
10^th^	Journal of Fungi	8.4	Multidisciplinary Digital Publishing Institute (MDPI)	9	116	12.89	7
10^th^	Chemistry and Biodiversity	3.5	John Wiley & Sons	9	65	7.22	4

* ACP, average citations per paper; TC, total citations; TP, total publications. Rank was based on TP.

This thematic and impact-driven stratification is further reinforced by the highly cited documents presented in **Table 2**, which collectively define the intellectual foundation and evolution of the field. Foundational reviews such as Lindequist et al. (2005) (910 citations) and Sullivan et al. (2006) (242 citations) establish the pharmacological and therapeutic relevance of medicinal mushrooms, serving as key conceptual anchors for subsequent research. Meanwhile, studies such as Reis et al. (2012) and Jayakumar et al. (2009) emphasize antioxidant activity and phenolic composition, reflecting the early dominance of functional and biochemical characterization. More recent highly cited works, including Bellettini et al. (2019) and Ruwizhi and Aderibigbe (2020), indicate a transition toward understanding cultivation factors, bioactive compound optimization, and molecular-level mechanisms, aligning with the growing interest in translational and applied research. Notably, the prominence of studies on immunomodulatory (Sarangi et al., 2006) and enzymatic/biodegradation functions (Palmieri et al., 2005) further demonstrates the expanding scope of *Pleurotus* research beyond nutrition into pharmaceutical and environmental applications.

**Table 2:** Highly cited studies shaping the development and evolution of *Pleurotus* spp. bioactivity research over 25 years.

Authors	Title	Year	Source title	Cited by	DOI
Lindequist et al. (2005)	The pharmacological potential of mushrooms	2005	Evidence-based Complementary and Alternative Medicine	910	10.1093/ecam/neh107
Bellettini et al. (2019)	Factors affecting mushroom *Pleurotus* spp.	2019	Saudi Journal of Biological Sciences	427	10.1016/j.sjbs.2016.12.005
Ruwizhi and Aderibigbe (2020)	Cinnamic acid derivatives and their biological efficacy	2020	International Journal of Molecular Sciences	415	10.3390/ijms21165712
Rathore et al. (2017)	Mushroom nutraceuticals for improved nutrition and better human health: A review	2017	PharmaNutrition	401	10.1016/j.phanu.2017.02.001
Reis et al. (2012)	Antioxidant properties and phenolic profile of the most widely appreciated cultivated mushrooms: A comparative study between in vivo and in vitro samples	2012	Food and Chemical Toxicology	311	10.1016/j.fct.2012.02.013
Sarangi et al. (2006)	Anti-tumor and immunomodulating effects of *Pleurotus ostreatus* mycelia-derived proteoglycans	2006	International Immunopharmacology	262	10.1016/j.intimp.2006.04.002
Palmieri et al. (2005)	Remazol Brilliant Blue R decolorization by the fungus *Pleurotus ostreatus* and its oxidative enzymatic system	2005	Enzyme and Microbial Technology	248	10.1016/j.enzmictec.2004.03.026
Sullivan et al. (2006)	Medicinal mushrooms and cancer therapy: Translating a traditional practice into Western medicine	2006	Perspectives in Biology and Medicine	242	10.1353/pbm.2006.0034
Raman et al. (2021)	Cultivation and Nutritional Value of Prominent *Pleurotus* spp.: An Overview	2021	Mycobiology	229	10.1080/12298093.2020.1835142
Cohen et al. (2014)	Chemical composition and nutritional and medicinal value of fruit bodies and submerged cultured mycelia of culinary-medicinal higher basidiomycetes mushrooms	2014	International Journal of Medicinal Mushrooms	223	10.1615/IntJMedMushr.v16.i3.80
Jayakumar et al. (2009)	*In-vitro* antioxidant activities of an ethanolic extract of the oyster mushroom, *Pleurotus ostreatus*	2009	Innovative Food Science and Emerging Technologies	223	10.1016/j.ifset.2008.07.002

### 3.4 Most productive authors and co-authorship networks

**Figure 4:**
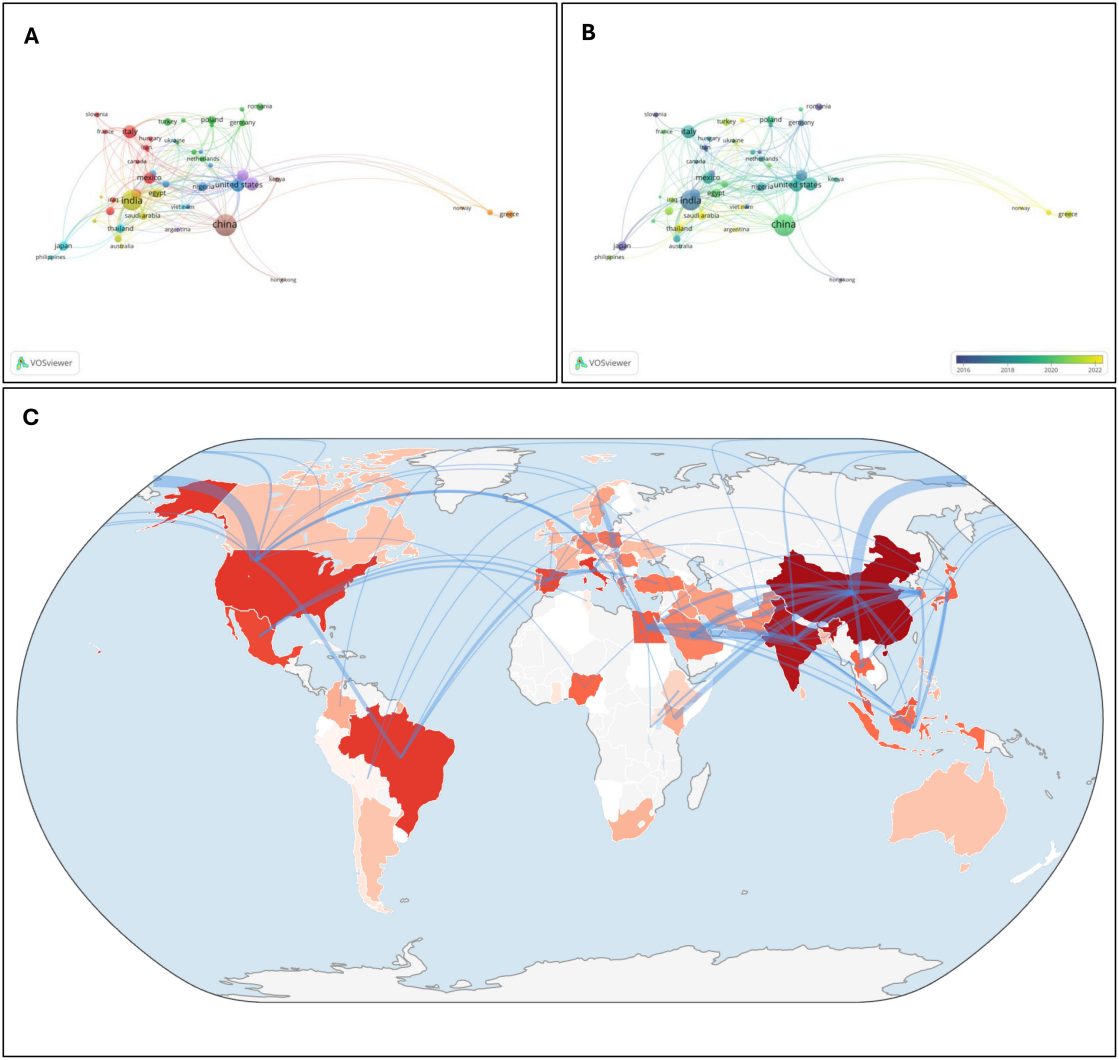
International collaboration patterns in *Pleurotus* spp. bioactivity research. **(A)** Country coauthorship network showing collaboration clusters among countries. **(B)** Overlay visualization illustrating the temporal evolution of international collaborations based on the average publication year. **(C)** World collaboration map depicting the geographic distribution and strength of international research collaborations.

Author productivity and co-authorship patterns reveal a highly interconnected yet regionally clustered research landscape in *Pleurotus* spp. studies. As shown in **Table 3**, Hu Qiu Hui (TP = 26; TC = 1,135; ACP = 43.65) emerges as the most productive and influential author, followed by Ma Gao Xing and Zervakis Georgios I., reflecting strong contributions from institutions in China and Europe. Notably, authors affiliated with Nanjing University and related Chinese institutions dominate in terms of publication output, while European researchers, particularly from Greece and Portugal, demonstrate competitive citation impact, as evidenced by high ACP values (e.g., Ferreira and Barros with ACP = 88.22). This indicates a dual pattern where Asian institutions lead in productivity, whereas European groups contribute highly cited and potentially more specialized or review-oriented work. Further insight into international collaboration patterns is illustrated by the country co-authorship network (**Figure 4A**), which identifies major collaboration clusters among countries. The temporal evolution of these collaborations is shown in the overlay visualization (**Figure 4B**), while the global collaboration map (**Figure 4C**) highlights the geographic distribution and strength of international research partnerships.

**Table 3:** Top 10 most relevant authors publishing *Pleurotus* spp.

Rank	Author	Affiliation	TP	TC	ACP	h-index
1^st^	Hu, Qiu Hui	Nanjing University	26	1,135	43.65	17
2^nd^	Ma, Gao Xing	Nanjing University	17	765	45.00	10
2^nd^	Zervakis, Georgios I.	Agricultural University of Athens	17	556	32.71	11
3^rd^	Koutrotsios, Georgios	Agricultural University of Athens	14	520	37.14	10
3^rd^	Zhao, Li Yan	Nanjing Agricultural University	14	685	48.93	12
4^th^	Yang, Wen Jian	Nanjing University	13	755	58.08	11
5^th^	Vamanu, Emanuel	Universitatea de Stiinte Agronomice si Medicina Veterinaria din Bucuresti	12	281	23.42	10
6^th^	Abdullah, Noorlidah	Universiti Malaya	11	334	30.36	8
6^th^	Kyriacou, Adamantini	Harokopio University of Athens	11	191	17.36	7
6^th^	Martinez, Angel T.	Centro de Investigaciones Biologicas Margarita Salas	11	581	52.82	10
7^th^	Cone, John W.	Wageningen University & Research	10	373	37.30	8
	Maiti, Tapas K.	Indian Institute of Technology	10	519	51.90	10
8^th^	Barros, Lillian	Instituto Politecnico de Braganca	9	794	88.22	9
	Ferreira, Isabel C.F.R.	Instituto Politecnico de Braganca	9	794	88.22	9
	Kerezoudi, Evangelia N.	Orebro Universitet	9	160	17.78	6
	Mitsou, Evdokia	Harokopio University of Athens	9	186	20.67	7
	Pei, Fei	Nanjing University	9	567	63.00	9
	Pletsa, Vasiliki	National Hellenic Research Foundation	9	146	16.22	6
	Saxami, Georgia	Harokopio University of Athens	9	151	16.78	7
9^th^	Angelini, Paola	Universita degli Studi di Perugia	8	202	25.25	7
	Hendriks, W.H.	Wageningen University & Research	8	317	39.63	6
	Iacomini, Marcello	Universidade Federal do Parana	8	198	24.75	6
	Jia, Le	Shandong Agricultural University	8	405	50.63	7
	Muszynska, Bozena	Jagiellonia University Medical College	8	212	26.50	5
	Xiao, Hang	University of Massachusetts Amherst	8	395	49.38	7
10^th^	Aguilar-Marcelino, Liliana	Instituto Nacional de Investigaciones Forestales, Agricolas y Pecuarias	7	57	8.14	4
	Heredia, Ana	Universitat Politecnica	7	133	19.00	5
	Isikhuemhen, Omoanghe S.	North Carolina Agricultural and Technical State University	7	54	7.71	4
	Ma, Ning	Nanjing University	7	456	65.14	6
	Siwulski, Marek	Uniwersystet Przyrodniczy w Poznaniu	7	106	15.14	5
	Venanzoni, Roberto	Universita degli Studi di Perugia	7	163	23.29	6
10^th^	Zhang, Jian Jun	Shandong Agricultural University	7	385	55.00	6

* ACP, average citations per paper; TC, total citations; TP, total publications. Rank was based on TP.

### 3.5 Co-occurrence network of *Pleurotus* research

**Figure 5:**
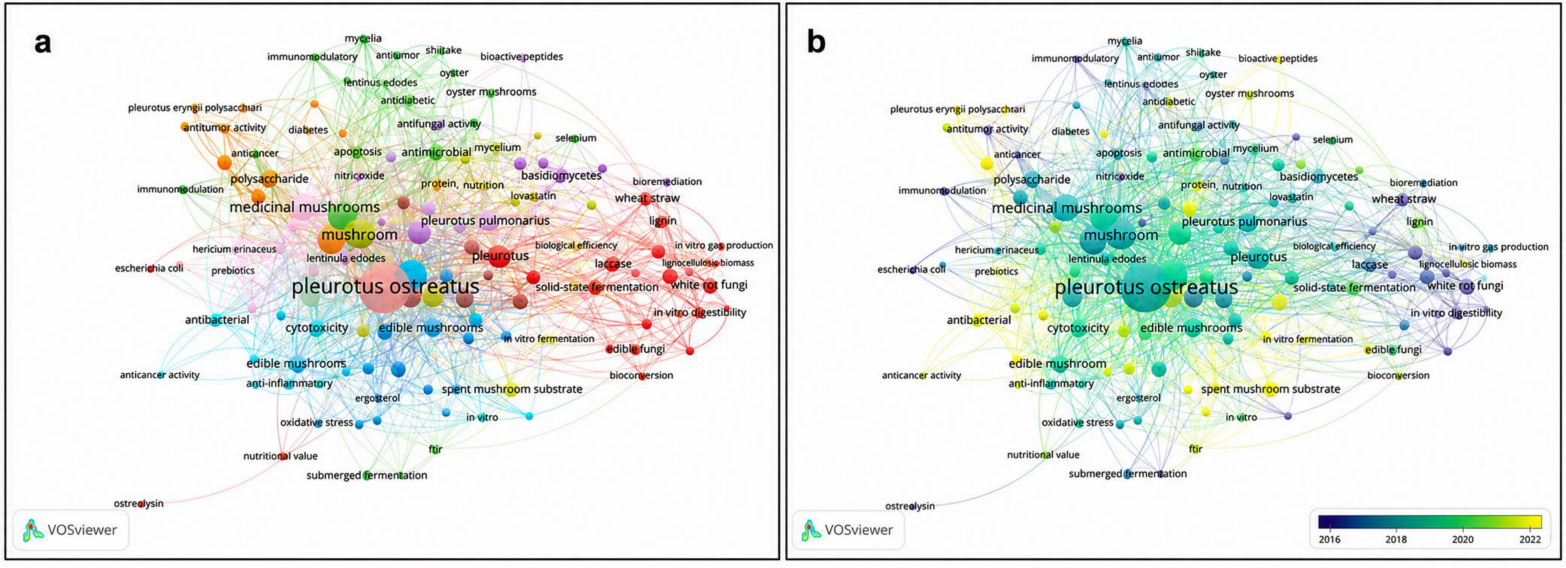
Keyword co-occurrence of research on *Pleurotus* spp. through **(a)** network visualization, and **(b)** overlay visualization.

Keyword co-occurrence analysis reveals a well-structured and thematically diverse research landscape in *Pleurotus* spp. studies, reflecting the field’s evolution from fundamental biology to multidisciplinary applications. As shown in **Figure 5**, the network visualization (a) identifies five major clusters, while the overlay visualization (b) highlights the temporal progression of research themes, indicating a shift from foundational studies toward more application-driven and mechanistic investigations. The red cluster is centered on core species such as *P. ostreatus*, emphasizing nutritional composition and phenolic content, thereby establishing the foundational knowledge of mushroom bioactivity. Closely linked, the blue cluster reflects therapeutic and medicinal applications, including antioxidant, antimicrobial, and cytotoxic activities, suggesting a strong translational bridge between food science and pharmacology. The green cluster further refines this direction by focusing on the isolation and characterization of bioactive compounds, underscoring the growing importance of molecular-level investigations in identifying functional metabolites. Meanwhile, the purple cluster highlights structural biochemistry, particularly polysaccharides such as β-glucans, and their roles in immune modulation and gut microbiota regulation, indicating an increasing focus on gut health and immunonutrition. In contrast, the orange cluster represents industrial biotechnology and mycoremediation, emphasizing enzymatic degradation, lignocellulosic processing, and environmental applications, thereby expanding the relevance of *Pleurotus* beyond health into sustainability domains. These thematic domains and their defining characteristics are summarized in **Table 4**, which further delineates the focus and notable features of each cluster.

**Table 4:** Thematic clusters in *Pleurotus* spp. bioactivity research. .

Cluster color	Theme	Focus	Notable features
Red	Core Species & Biological Activity	Centers on *P. ostreatus*, examining nutritional and chemical profiles, including phenolic compounds.	Emphasizes health-promoting properties of primary cultivars; highlights nutraceutical potential of staple edible mushrooms.
Blue	Therapeutic & Medicinal Applications	Bridges food and medicine, exploring antimicrobial, antioxidant, and cytotoxic activities.	Highlights therapeutic applications and clinical relevance; positions species for pharmacological and medicinal studies.
Green	Bioactive Compounds & Supplements	Focuses on isolating and characterizing bioactive molecules.	Defines compounds responsible for health benefits; links molecular identification to nutraceutical applications.
Purple	Structural Biochemistry & Gut Health	Investigates mushroom polysaccharides like β-glucans, their chemical structure and functional role.	Connects compounds to immune modulation and gut microbiota; emphasizes digestive and prebiotic health effects.
Orange	Industrial Biotechnology & Mycoremediation	Studies enzymatic degradation of lignin and other materials.	Demonstrates industrial and environmental potential in waste management and bioenergy; focuses on fermentation and bioremediation.

Analysis at the species level indicates that *Pleurotus ostreatus* is the most extensively studied taxon within the genus. This species is a focal point of research on nutritional composition, antioxidant activity, polysaccharides, immunomodulation, cultivation, and environmental biotechnology, underscoring its significance as both a commercially cultivated mushroom and a model organism in Pleurotus studies. Other frequently examined species include *P. eryngii*, *P. pulmonarius*, *P. sajor-caju*, *P. florida*, and *P. citrinopileatus*, which have been studied for their bioactive compounds, therapeutic potential, and industrial applications. However, the current literature reveals a pronounced taxonomic focus on a limited number of commercially cultivated species, indicating that the bioactive and biotechnological potential of many lesser-known *Pleurotus* taxa remains underexplored.

### 3.6 Emerging frontiers in *Pleurotus* spp. bioactivity research

**Figure 6:**
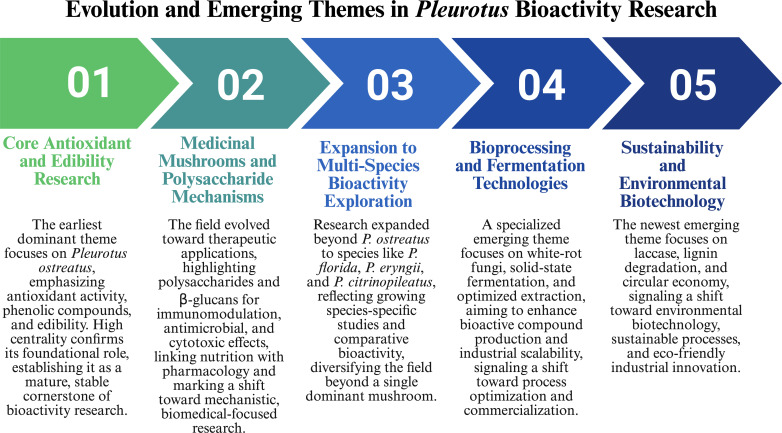
Emerging research frontiers in *Pleurotus* bioactivity studies.

Emerging frontiers in *Pleurotus* spp. bioactivity research reflects a pronounced transition from descriptive and antioxidant-centered investigations toward technologically integrated, application-driven, and sustainability-oriented frameworks. As illustrated in **Figure 6**, contemporary research is increasingly characterized by the convergence of bioactivity studies, industrial biotechnology, and environmental sustainability, indicating a shift toward systems-level approaches aligned with global bioeconomy priorities. While early investigations predominantly focused on *P. ostreatus* and its nutritional and antioxidant properties, recent studies have expanded both taxonomic and mechanistic scope, incorporating underexplored species such as *P. citrinopileatus* and *P. florida*, alongside more targeted evaluations of molecular pathways underlying bioactivity. This transition is further contextualized through the temporal framework presented in **Table 5**, which reveals not only chronological progression but also a functional transformation of the field. During the foundational era (2000-2010), research primarily centered on optimizing cultivation strategies, particularly solid-state fermentation on agro-wastes, and establishing baseline enzymatic and biochemical profiles, with applications largely confined to nutritional use and preliminary antimicrobial and antitumor assessments. In the diversification era (2011–2019), the field underwent significant methodological and application-oriented expansion, driven by the adoption of green extraction technologies and submerged fermentation systems, which enabled more efficient production and characterization of bioactive compounds. This period also marked a transition toward targeted therapeutic applications, including immunomodulatory, anti-diabetic, and anti-inflammatory interventions, alongside the early emergence of cosmeceutical and peptide-based products.

**Table 5:** Evolution of *Pleurotus* bioactivity research (2000-2025).

Period	Technological and methodological advances	Industrial applications	Sustainability initiatives
2000-2010 *(Foundational Era)*	Basic solid-state fermentation (SSF) on agro-wastes (straw, husks); optimization of cultivation parameters and spawn production; early enzyme characterization (laccase, peroxidase)	Primarily used as a nutritious food source; early nutraceutical exploration (polysaccharide extracts); crude extracts studied for antimicrobial and antitumor activity	Limited agro-waste utilization; initial reuse of spent mushroom substrate (SMS) as animal feed
2011–2019 *(Era of Diversification)*	Advanced SSF targeting enzyme production; green extraction technologies (UAE, MAE); submerged fermentation (SmF) for mycelial biomass and bioactive compound production	Targeted health applications including immunomodulatory, anti-diabetic, and anti-inflammatory products; early cosmetic formulations; bioactive peptide production from protein hydrolysates	Valorization of diverse agro-industrial wastes (coffee husks, oil cakes); SMS applied in mycoremediation and as biofertilizer
2020-2025 *(Circular Bioeconomy Era)*	AI-assisted process optimization (ANN-GA); synergistic extraction technologies (plasma-ultrasound); biorefinery integration; development of mycelium-based composites and nanoparticle platforms	Precision nutraceuticals for anti-aging and cognitive health; cosmeceuticals for anti-photoaging; active food packaging; functional biomaterials for tissue engineering and drug delivery	Integrated waste valorization systems: SMS used for biopesticides, animal feed, and substrate recycling; mycelium-based biocomposites and upcycling of complex agro-industrial wastes (brewer’s spent grain, MAP residues)

The most recent phase, defined here as the circular bioeconomy era (2020-2025), represents a paradigm shift toward integrative and high-technology-driven research. Advances such as AI-assisted process optimization, biorefinery integration, and synergistic extraction technologies have enabled more precise and scalable production of bioactive compounds and functional biomaterials. Concurrently, applications have evolved toward high-value outputs, including precision nutraceuticals, active food packaging systems, and biomaterials for tissue engineering and drug delivery. Sustainability has likewise transitioned from a peripheral consideration to a central research driver, with integrated waste valorization systems, mycelium-based composites, and the upcycling of complex agro-industrial residues reflecting a circular systems approach. Collectively, these developments indicate that *Pleurotus* research is increasingly positioned within an interconnected framework that links molecular bioactivity, industrial scalability, and environmental sustainability, although the parallel progression of these domains suggests that full integration remains incomplete and warrants further interdisciplinary consolidation.

## 4 Discussion

Over the past 25 years (2000-2025), *Pleurotus* bioactivity research has evolved from fragmented, descriptive investigations into a more structured and increasingly interdisciplinary field. This progression is reflected in the divergence between publication output and citation impact (**Figure 2**), where relatively stable publication trends contrast with a marked increase in citations after 2012, suggesting a shift toward greater scientific influence and consolidation of knowledge. Early studies were largely exploratory, focusing on taxonomic classification, cultivation practices, and compositional profiling, which established *Pleurotus* as both a nutritionally valuable and biologically relevant genus (Lindequist et al., 2005; Sullivan et al., 2006). However, the transition toward more mechanistic and application-driven research indicates that the field has moved beyond descriptive characterization toward a more mature phase aligned with contemporary natural products research.

The geographic distribution of research highlights a dual structure in global knowledge production. While countries such as China and India dominate in publication output (**Figure 3**), citation influence appears more globally distributed, with European and North American studies contributing disproportionately to conceptual and highly cited work (Sarangi et al., 2006; Cohen et al., 2014). This divergence reflects differences in research infrastructure, funding capacity, and publication strategies, where high-output regions tend to prioritize experimental productivity, while others contribute integrative and methodological advancements. Importantly, this pattern suggests that scientific impact in *Pleurotus* research is shaped not only by biological resource availability but also by institutional and collaborative dynamics.

Collaboration networks further reveal a structurally complex and partially fragmented research landscape. Co-authorship patterns (**Figure 4**) demonstrate strong regional clustering alongside emerging international linkages, indicating that while collaboration has increased, it remains unevenly distributed. Research communities continue to be organized around specific thematic domains, including polysaccharide chemistry, enzymology, pharmacological bioactivity, and environmental biotechnology (Palmieri et al., 2005; Reis et al., 2012). Although bridging authors and institutions are beginning to connect these clusters, the persistence of partially isolated sub-networks suggests that integration between mechanistic laboratory research and clinically or industrially oriented applications remains limited. This structural fragmentation may contribute to the observed gap between scientific discovery and real-world implementation.

Thematic evolution analysis (**Figure 5**) provides further evidence of this transition, showing a clear shift from early descriptive studies toward molecular and mechanistic investigations. Initial research themes centered on protein composition, mineral content, and cultivation parameters have progressively evolved toward more complex topics such as oxidative stress regulation, immune modulation, and cellular signaling pathways (Lindequist et al., 2005; Bellettini et al., 2019; Ruwizhi and Aderibigbe, 2020). Despite this evolution, core themes, particularly polysaccharides, antioxidant activity, and immunomodulatory effects, remain central to the field (Rathore et al., 2017; Reis et al., 2012), reflecting both their therapeutic relevance and their methodological accessibility. At the same time, emerging areas such as environmental biotechnology and metabolic health research highlight the expanding role of *Pleurotus* spp. in addressing interconnected challenges in human health and environmental sustainability (Palmieri et al., 2005; Raman et al., 2021). This convergence aligns with a One Health perspective, although the continued separation of biomedical and environmental research domains suggests that full interdisciplinary integration has not yet been achieved.

Despite the strong growth and intellectual maturation of the field, a substantial translational gap persists between laboratory discoveries and real-world applications. Highly cited studies continue to emphasize foundational bioactivity, methodological development, and integrative reviews (Lindequist et al., 2005; Reis et al., 2012; Bellettini et al., 2019), whereas relatively few investigations progress toward clinical validation or large-scale industrial implementation (Cohen et al., 2014; Raman et al., 2021). This imbalance reflects persistent barriers, including limited pharmacokinetic data, lack of standardized extraction and characterization protocols, and challenges in scalability and regulatory alignment (Palmieri et al., 2005). As a result, citation impact often reflects academic visibility rather than direct societal or clinical relevance. Addressing these limitations will require stronger integration between experimental research, clinical validation, and industrial application, supported by interdisciplinary collaboration and translational research frameworks that bridge the gap between discovery and implementation.

### 4.1 Translational bottlenecks and future directions in *Pleurotus* research for human clinical application

**Figure 7:**
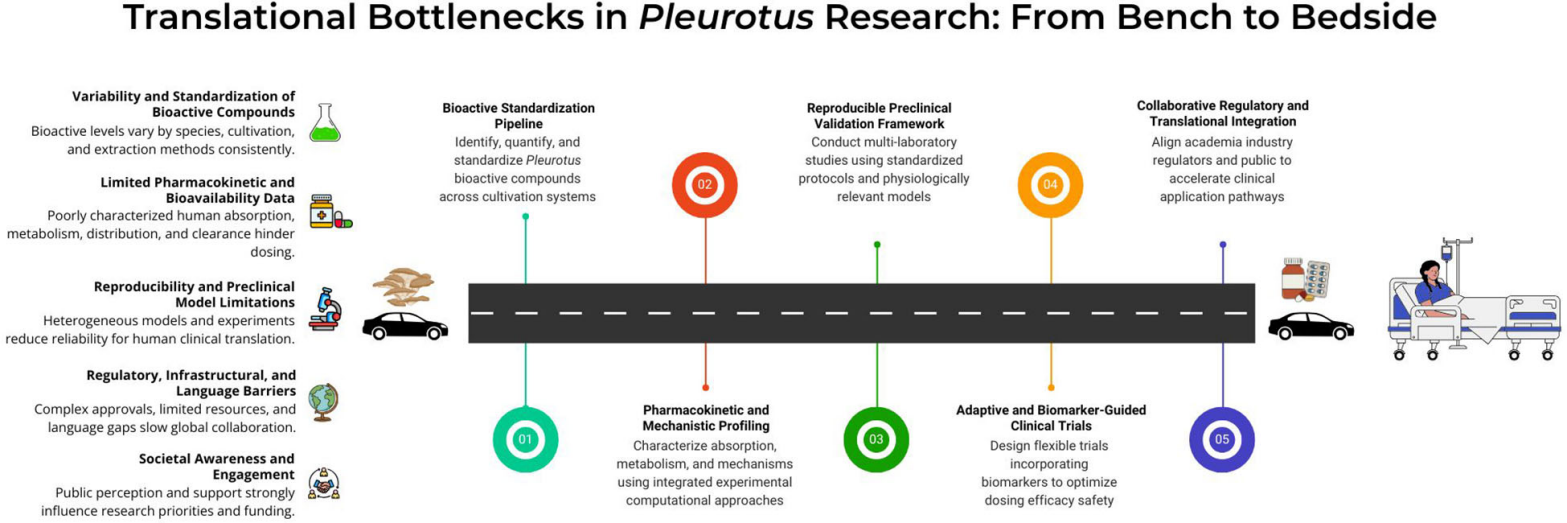
Translational bottlenecks and future directions in *Pleurotus* spp. research for human clinical application.

*Pleurotus* species, rich in β-glucans, proteo-polysaccharides, and phenolic compounds, exhibit significant therapeutic potential, including immunomodulatory, antioxidant, and antitumor activities; however, translation into human clinical application remains constrained by interconnected scientific, technical, and systemic bottlenecks. As illustrated in **Figure 7**, variability in bioactive composition driven by species differences, cultivation conditions, and extraction techniques represents a primary limitation, complicating standardization, reproducibility, and the establishment of consistent therapeutic outcomes and dosing strategies (Khatua, 2025; Selvaananthi and Beulah Jerlin, 2024; Araújo et al., 2025). This variability is further exacerbated by taxonomic ambiguity, where phenotypic plasticity and genus-level classification obscure species-specific pharmacological profiles, necessitating molecular authentication tools such as ITS sequencing and multi-locus markers, alongside chemotaxonomic profiling (LC-MS, HPLC) to ensure biochemical consistency and mechanistic clarity (Li et al., 2022; Sharma et al., 2020; Zervakis, 2004; He et al., 2017; Li et al., 2017; Flores et al., 2023; Ćilerdžić and Galić, 2025; Acharya et al., 2017).

Pharmacokinetic limitations further hinder clinical translation, as human data on absorption, distribution, metabolism, and excretion remain scarce, and bioavailability is often unpredictable due to solubility and stability constraints of compounds such as β-glucans and ergothioneine (Araújo et al., 2025; Ioannidis, 2019; Stegemann et al., 2007; Zhang et al., 2024; de Oliveira et al., 2022). Reproducibility is similarly constrained by heterogeneous experimental designs, limited multi-center validation, and preclinical models that inadequately reflect human physiological complexity (Karp, 2018; Fell et al., 2022; Marshall et al., 2005; Yao and Kanellakis, 2020). Although emerging extraction approaches such as aqueous two-phase systems and enzyme-assisted extraction enhance recovery efficiency, the absence of standardized protocols continues to limit cross-study comparability (Yang et al., 2025; Xie et al., 2024; You et al., 2025).

Regulatory, economic, and societal factors further shape translational outcomes. Natural-product therapeutics require rigorous clinical validation and mechanistic evidence, yet fragmented funding, limited infrastructure, and unclear regulatory pathways delay development and commercialization (Letourneur et al., 2021; Balash et al., 2025; Kanwal et al., 2025; Lems et al., 2025; Araújo et al., 2025). Public perception and awareness also influence research prioritization and adoption, emphasizing the socio-scientific dimension of translation. Additionally, structural inequalities in global research limit participation from biodiversity-rich regions due to disparities in funding, infrastructure, and access to international networks, reinforcing a core–periphery knowledge structure (Valdez et al., 2024; Singh et al., 2025; Roch et al., 2025; Liu et al., 2017; He et al., 2020, He et al., 2021). Practices such as parachute science and tokenistic collaborations further marginalize local expertise, necessitating equitable partnerships, inclusive funding systems, and open science frameworks to foster global research integration (Koch et al., 2025; Doğan et al., 2023; Tolochko and Vadrot, 2021; Coombe et al., 2023; Bezuidenhout, 2025; Báldi and Palotás, 2021; Klincewicz and Esmaeil Zaei, 2025; Elkayam Cohen and Fluhr, 2024; Roberts et al., 2012).

Integrating *Pleurotus* research within a One Health framework introduces additional challenges, particularly in scaling environmental applications such as biodegradation and agro-industrial waste valorization from laboratory to field settings. Standardization of environmental endpoints, ecotoxicological validation, and long-term monitoring remain underdeveloped, while weak governance structures and limited interdisciplinary coordination hinder implementation (Lozada-Martinez et al., 2026; Faijue et al., 2024; Pumipuntu and Suwannarong, 2025; Abass et al., 2025; Utomo, 2026; Meisner et al., 2024; Nowbuth and Parmar, 2025; Garg et al., 2025). Similarly, industrial-scale deployment is constrained by low technological readiness levels (TRL 3-5), high operational costs, and insufficient policy support, despite strong potential in enzyme production, lignocellulosic bioconversion, and circular bioeconomy applications (Schein et al., 2026; Bonis et al., 2026; Mastranzo-Pérez et al., 2025; Ghafoor and Niazi, 2025; Ayuso et al., 2025; Morenikeji Abel et al., 2025; Silva et al., 2024).

Emerging integrative strategies provide viable pathways to overcome these barriers. Omics-based approaches and systems biology enable precise identification, monitoring, and mechanistic understanding of bioactive compounds (Patel et al., 2017; Govindaraj and Renganathan, 2017; Ashmore-Harris et al., 2024), while in silico pharmacokinetic modeling and non-invasive sampling techniques improve dose optimization and safety in clinical studies (Ashmore-Harris et al., 2024; Desai and Cristofoletti, 2025). Adaptive clinical trial designs and multi-laboratory validation frameworks can enhance reproducibility and translational relevance (Karp, 2018; Wang and Wang, 2024; Lippi et al., 2016), whereas coordinated governance, policy engagement, and community-based initiatives are essential to bridge research, industry, and societal adoption (Lozada-Martinez et al., 2026; Karakhanova et al., 2024; Delonge et al., 2020; Gómez-Jiménez et al., 2026).

### 4.2 Limitations of the study

Despite the comprehensive scope and analytical depth of this scientometric assessment, several methodological limitations must be acknowledged. First, the reliance on a single bibliographic database (Scopus) introduces inherent database bias, as publication coverage, indexing criteria, and journal inclusion policies differ across platforms. This may lead to the underrepresentation of relevant studies indexed in other databases such as Web of Science, PubMed, or regional repositories, particularly those originating from non-English-speaking regions where *Pleurotus* cultivation and application are highly prevalent. Additionally, citation-based indicators, while widely used as proxies for scientific impact, are subject to citation bias and may not accurately reflect translational relevance or real-world application. Highly cited studies often correspond to foundational or review articles, whereas applied research and community-based innovations may remain underrepresented despite their significant socioeconomic contributions.

Furthermore, the use of author keywords and indexed terms for co-occurrence and thematic analyses introduces limitations related to keyword normalization and semantic variability. Inconsistent terminology, broad descriptors such as *Pleurotus* spp., and variations in keyword assignment may reduce species-level resolution and obscure biochemical and pharmacological differences across taxa. This constraint is particularly relevant in a biologically diverse genus where functional properties vary substantially between species. Finally, although this study incorporates a translational perspective, the evaluation of clinical and industrial progression is inferred from publication trends rather than directly validated through clinical trial data, regulatory frameworks, or technology readiness assessments. Future studies integrating multi-database approaches, standardized keyword harmonization, and complementary systematic or qualitative methodologies may further enhance the robustness and translational relevance of scientometric analyses in *Pleurotus* research.

## 5 Conclusion

This scientometric analysis presents a comprehensive overview of global research trends concerning the bioactivity and pharmacological potential of *Pleurotus* species from 2000 to 2025. The results demonstrate a gradual shift in the field from primarily descriptive and nutrition-focused studies to research emphasizing molecular bioactivity, biotechnology, and sustainability applications. Research activity was predominantly concentrated in Asia, especially in China and India, whereas citation influence was more widely distributed across Europe and North America. Thematic analysis reveals increasing interest in functional foods, bioactive compounds, industrial biotechnology, and environmental applications. However, the majority of existing literature remains centered on preclinical and experimental studies, with relatively limited clinical validation and large-scale implementation. There are ongoing opportunities to enhance integration among biomedical, industrial, and sustainability-focused research domains. Advancing the field may require continued interdisciplinary collaboration, methodological standardization, and translational research. In summary, this study elucidates the development of *Pleurotus* bioactivity research over the past 25 years and identifies emerging themes and future research directions.

## Data Availability

The original contributions presented in the study are included in the article/supplementary material. Further inquiries can be directed to the corresponding author.
